# Outcome and Challenges of Kidney Transplant in Patients with Sickle Cell Disease

**DOI:** 10.1155/2013/614610

**Published:** 2013-04-11

**Authors:** U. H. Okafor, E. Aneke

**Affiliations:** Renal Unit, Department of Medicine, Enugu State University Teaching Hospital Parklane, Enugu, Nigeria

## Abstract

Sickle cell nephropathy is a common presentation in patients with sickle cell disease. End-stage kidney disease is the most severe presentation of sickle cell nephropathy in terms of morbidity and mortality. Sickle cell disease patients with end-stage kidney disease are amenable to renal replacement therapy including kidney transplant. Kidney transplant in these patients has been associated with variable outcome with recent studies reporting short- and long-term outcomes comparable to that of patients with HbAA. Sickle cell disease patients are predisposed to various haematological, cardiorespiratory, and immunological challenges. These challenges have the potential to limit, delay, or prevent kidney transplant in patients with sickle cell disease. There are few reports on the outcome and challenges of kidney transplant in this group of patients. The aim of this review is to highlight the outcome and challenges of kidney transplant in patients with sickle cell disease.

## 1. Introduction

Sickle cell disease (SCD) is a haematological disorder associated with multisystemic complications and manifestations [1]. There had been significant improvement in the outlook of adults with sickle cell disease. The Cooperative Study of Sickle Cell Disease (CSSCD) and other observational studies had helped to define the prognosis and common complications that occur as the patient ages. Improvement in management of infections and central nervous system (CNS) complications in childhood, active health maintenance for adults, new interventions, and improved psychosocial support have all contributed to a reduction in morbidity and mortality. More than 90 percent of patients of all phenotypes will survive for more than 20 years, and significant numbers are older than age of 50 years [1]. Thus, chronic and long-term complications associated with SCD including sickle cell nephropathy/end stage renal disease (ESRD) are becoming common. 

Treatment of sickle cell nephropathy is fraught with many challenges and has variable outcome. Renal replacement therapy is required in patients with uraemia, circulatory overload, pulmonary oedema, and ESRD. Kidney transplant is the treatment of choice for eligible patients with ESRD. However, kidney transplant is not readily available in most of the developing countries because of cost, poverty, few transplant centres, and lack of donors [2]. SCD and prevailing complications worsen these challenges, further limiting kidney transplant in these patients. 

The aim of this study is to review the available literature highlighting the outcome and challenges of kidney transplant in patients with sickle cell disease. The available studies and reviews were assessed using the search terms kidney diseases and sickle cell disease, kidney transplant and sickle cell disease, surgery and sickle cell disease, anaesthesia and sickle cell disease, and complications of sickle cell disease.

## 2. Sickle Cell Nephropathy (SCN)

SCN is a functional and structural abnormality seen in patients with sickle cell disease. It is most pronounced in homozygous HBSS patients, but had been reported in patients with HbAS, HbSC, HbSD, HbSE, and HbSthal [3]. SCN had been reported to be far less frequent and severe in HbSC and HbAS than other phenotypes [4, 5]. Hyperfiltration assessed by estimated GFR is found in only 5%, albuminuria in 7%, and chronic renal failure in 2% of young HbSC patients compared with 51%, 59%, and 7%, respectively, in SCA patients [5]. Sickle cell-associated glomerulopathy is also rarely encountered in HbSC patients. However, further data are needed to address the issue as to whether HbSC may be an additional risk factor for chronic kidney diseases from other causes, as recently suggested for patients with sickle cell trait [4]. 

Twenty-five to thirty percent of SCD patients were reported to have proteinuria, and 5–18% of them develop renal failure [6, 7]. SCN constitute 0.11% of ESRD reported in the United States renal data survey (USRDS); 93% of them were African American. SCD was documented as risk factor for development of ESRD [5]. The mean age of the patient was 23 to 40 years, and survival was found to be 4 years [3, 8, 9]. 

Several mechanisms had been proposed as cause of various glomerular and tubular changes in patients with SCN. These proposed mechanisms include glomerular and tubular ischaemia, iron overload and subsequent deposition in the kidneys, continued intracapillary fragmentation and phagocytosis of sickled red cells, immune complex formations, FSGS associated with glomerular hyperfiltration, and/or intrinsic glomerular capillary injury [10].

**Figure 1:**
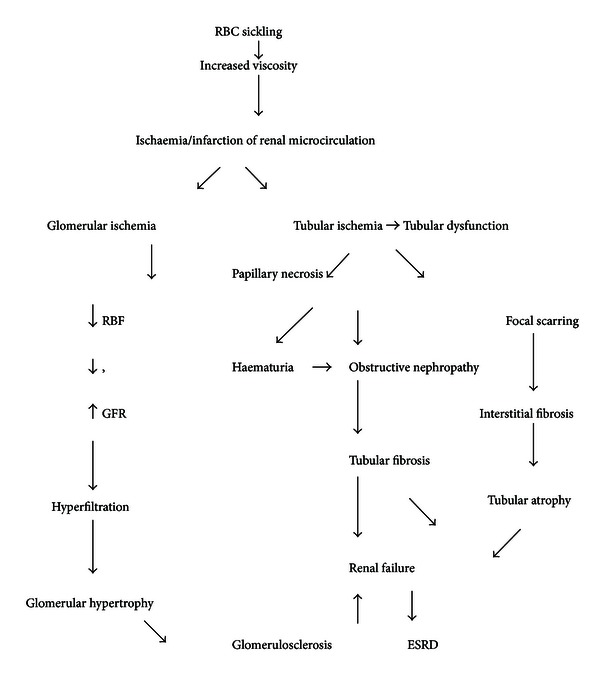
Schematic diagram of pathophysiology of SCN.

The pathophysiology of SCN (Figure 1) is related to the normal renal medullar environment which is characterised by low oxygen tension, low pH, and high osmolality. These conditions in SCD patients predispose to red blood cell sickling, increased blood viscosity leading to ischemia, and eventual infarction of renal microcirculation. Glomerular ischemia leads to compensatory increase in renal blood flow and glomerular filtration rate (GFR). The resulting hyperfiltration, combined with glomerular hypertrophy, probably contributes to glomerulosclerosis. As glomerulosclerosis becomes more extensive, the GFR starts to decrease and nonselective proteinuria may result leading to chronic kidney disease and subsequently ESRD [11–13]. Red blood cell (RBC) sickling in the vasa rectae is believed to interfere with the countercurrent exchange mechanism in the inner medulla. The resulting impairment of free water resorption manifests clinically as nocturia or polyuria [14, 15].

Furthermore, ischemia involving the renal medulla will also lead to the inability to maintain a hydrogen ion gradient (causing an incomplete form of distal renal tubular acidosis) and an electrochemical gradient (leading to hyperkalemia) along the collecting ducts. Gross hematuria can be secondary to papillary necrosis resulting from medullary ischemia and infarction. The sloughed papillae may obstruct urinary tract outflow leading to obstructive nephropathy and consequently renal failure [16]. 

The gross appearance of kidneys in patients with SCN initially is either normal size or enlarged. Subsequently, as the SCN progresses, the kidneys are shrunken. The histological abnormalities noted in these patients include glomerular enlargement, haemosiderin deposit, papillary necrosis, cortical infarction, focal segmental glomerulosclerosis, membranoproliferative glomerulonephritis, tubular atrophy, and interstitial fibrosis [14, 17, 18].

Proteinuria is an early sign of SCN, increasing with age, and is a predictor of progression of SCN. Proteinuria is more commonly encountered in patients with homozygous (hemoglobin SS) sickle cell disease than in other hemoglobinopathies [6]. Patients with HbSC had also been noted to develop renal insufficiency later than patients with HbSS [19]. A prospective study by Powars et al. showed that severe anaemia, hypertension, proteinuria, nephrotic syndrome, and microscopic hematuria were found to be significant predictor of chronic renal failure [20]. In addition, it has been shown that there is a high degree of association between proteinuria, chronic renal insufficiency, and increasing age. 

## 3. Outcome of Kidney Transplant in SCD

SCD patients with end-stage renal disease can be treated with both hemodialysis and peritoneal dialysis. Survival of patients on hemodialysis has been shown to be equivalent to other nondiabetic ESRD patients [21]. SCD patients with ESRD can also undergo kidney transplantation. The outcome of kidney transplant in these SCD patients is variable (Table 1). Earlier reports on kidney transplant in SCD patients suggested poor allograft survival and other disease-specific problems [22–24]. However, later studies have reported graft and patient survival rates comparable to those of nondiabetic patients with normal haemoglobin genotype [25–30]. A more recent study of renal transplantation in SCD reported short-term patient and allograft outcomes comparable to other age-matched African Americans [8].

There was a trend toward improved survival in those SCD patients who received transplants compared to those on chronic dialysis with one-year patient and graft survival of 87% and 67%, respectively [24]. However, there was a shorter cadaveric graft survival and high risk of graft loss with longer followup in the SCD patient group [31]. In an update registry in 1987, data recollected from 45 renal transplants performed in 40 recipients revealed a one-year patient survival rate of 88% and graft survival rate of 82% in living donor transplant recipients; however, one-year patient and graft survival rate was 62% in cadaveric transplant recipients [26].

Ojo and colleagues [31] reported that short-term survival of renal allograft in recipients with end-stage SCN was similar to that achieved in patients with other causes of end-stage renal disease, comparatively diminished long-term outcome and better patient survival with renal transplantation relative to dialysis in end-stage SCN. They further reported delayed graft function and acute graft rejection in 24% and 26% of patients, respectively. Warady and Sullivan [29] published the outcome for 9 patients on the pediatric register that underwent kidney transplant. They reported good graft survival rates of 89% and 71% at 1 and 2 years. For the 3 patients whose grafts eventually failed, 1 had acute rejection, 1 died from unrelated cause, and 1 had chronic rejection. Also, various studies had reported recurrent nephropathy in the allograft kidneys [27, 29, 31, 32].

In our study [35], we reported that SCD patient improved remarkably following kidney transplant. There was no operative or postoperative patient or graft related complication. At a year posttransplant, the graft was structurally and functionally normal; the patient was clinically stable, had normal quality of life, and the blood pressure and haematological parameters were within normal range. Ten-year survival had also been reported as 56% by Scheinman in his review of sickle cell disease and the kidney [33]. 

Brennan and associates [36] proposed a protocol after a successful living-unrelated transplant at their center. They suggested preoperative transfusion until sickle cell preparation is negative, use of antithymocyte globulin on induction, and use of hydroxyurea in preference of azathioprine with the dual purpose of immunosuppression and stimulation of fetal hemoglobin [36]. In a review by Scheinman [37], warming the graft with saline at 37°C, along with infusion of dopamine at 4 *μ*g/kg/min during and after transplant, was advocated. Other suggestions include extraintravenous fluid to decrease blood viscosity, supplemental oxygen, and recombinant erythropoietin until autoproduction is sufficient. For patients who develop sickle crises, IV fluid and partial exchange transfusions also have been suggested [9].

The recommendations for perioperative management of kidney transplant in patients with SCD to enhance better outcome include the following.

- Ensure the operating and anesthesia teams are aware of the diagnosis of a sickle cell syndrome and the need for special attention in the patient.
- Ensure that patients preoperative haemoglobin of 10 g/dL is achieved.
- Reduce hyperviscosity especially in patients with SCD-SC; there may be a need for exchanging blood transfusion.
- Minimize alloimmunization by giving antigen-matched blood.
- Preoperative monitoring of intake and output, hematocrit, peripheral perfusion, and oxygenation status.
- Intraoperative monitoring of blood pressure, cardiac rhythm and rate, and oxygenation.
- Intensive postoperative care including attention to hydration, oxygen administration with careful monitoring, and respiratory therapy.
-  Scrupulous followup of patients with stringent monitoring of patients wellbeing, renal function, drug level, and so forth.

Thus, sickle cell disease or trait should not be regarded as a contraindication to transplantation, at least from the patient and allograft survival standpoint.

## 4. Challenges of Kidney Transplant in Sickle Cell Disease Patient

Despite encouraging results on survival advantage of renal transplantation over maintenance hemodialysis, renal transplantation in sickle cell disease is used less frequently, possibly reflecting the limited experience with renal transplantation and/or various challenges associated with kidney transplant in this patient population [23]. Patients with SCD are prone to various organ dysfunctions which predispose them to haematologic, cardiovascular, pulmonary, and immunologic complications. These complications influence and compromise the choice, fitness, and outcome of kidney transplant in these patients. This clinical sequel could manifest during pre, intra- and postoperative managements of these patients.

## 5. Haematological Challenges

The hallmark of sickle cell anaemia is the presence of sickle cells in the peripheral blood and propensity to recurrent intravascular occlusion, haemolysis, and endemic anaemia, predisposing the patient to various severities of tissue hypoxia/ischaemia. General anaesthesia and various anaesthetic drugs have the potential to worsen this hypoxic state and can precipitate vasoocclusive crises intra- or immediate postoperative period [38–42]. Dehydration resulting from fluid deprivation, excessive fluid loss, and inability to ingest fluids had been noted to occur earlier in patients with SCD, thus exposing them to the additional risk of potential sickle cell crisis and acute kidney graft injury [43]. 

An increase in frequency of crisis has also been demonstrated in patients who underwent a successful kidney transplantation [23–25, 30]. This was attributed to improved erythropoeis resulting from restoration of erythropoitein level by the kidney transplant. Renal infarction, a probable secondary consequence of Hb S polymerization, cell sickling, and vaso occlusion, has been reported to occur as early as 6 days following transplantation [44]. Furthermore, there has been report of recurrent sickle cell nephropathy following kidney transplant, and this may had resulted from the heightened vaso occlusion of the transplant kidney microcirculation [10, 23]. Kim et al. [34] recently reported loss of allograft function following intragraft vasoocclusive crises in sickle cell disease patient.

Various suggestions for risk reduction in SCD patients undergoing surgery have been made, including correction of anemia by simple or exchange blood transfusion, attention to hydration and oxygenation, postoperative respiratory care, and selection of less aggressive or extensive surgical procedures. The protocol specified a minimum of 8 hours of preoperative hydration, with intraoperative monitoring of temperature, blood pressure, electrocardiographic features, and oxygenation. These had been reported to reduce the frequency of sickling and vasoocclusive crises in these SCD patients [45]. 

Bone marrow transplantation has recently emerged as a novel treatment for SCA. There had been reports suggesting a beneficial effect of bone marrow transplantation in improving target organ damage, including chronic lung, bone, and central nervous system disease. Whether bone marrow transplantation in the early stage of the disease can reverse or halt the progression of established sickle cell nephropathy is unknown and awaits clinical studies. Furthermore, experience in stem cell and kidney transplant is lacking; however, it may have potential in improving the long term outcome of kidney transplant in sickle cell patients [46, 47].

## 6. Cardiovascular Challenges

The perennial anaemia and recurrent vasoocclusive phenomenon prevalent in SCD patients predispose them to various cardiovascular morbidity and mortality. Data indicate that cardiomegaly resulting from recurrent anaemia, and myocardial ischemia resulting from combined effect of anaemia, microthrombi, bone marrow embolism, and increased blood viscosity is a common presentation in SCD. These conditions predispose the patients to various myocardial dysfunctions manifesting as tachycardia, hyperactive precordium, displaced apex, systolic murmur, and occasionally premature heart beats [48, 49]. Sickle cell disease patients have also been noted to have higher blood pressure than their counterpart with normal haemoglobin (HbAA) [50]. In a study of cardiac function in 200 SCD patients using echocardiogram, patients were reported to have increased biventricular and left atrial chamber dimensions and increased interventricular septal thickness [51]. 

These cardiovascular complications are further heightened by the presence of kidney disease with the associated negative impact on the heart and vessels. These cardiovascular complications in SCD patients with ESRD result from fluid overload, hyperparathyroidism, electrolyte imbalance, anaemia, hypertension, malnutrition, dyslipidaemia, and atherosclerosis. Kidney transplant being a major surgical procedure further worsens cardiovascular function in SCD patients [45]. 

Postoperative care of these patients is greatly influenced by the stability of the cardiovascular system. These patients are at risk of fluid overload, drug toxicity, and immunologic conditions that can impact negatively on the heart and circulatory system thus perpetuating the cardiovascular morbidity. This cardiovascular threat can be reduced by careful selection of anaesthetic agent, strict monitoring of fluid balance, haematocrit of at least 30%, and adequate intraoperative oxygenation [45].

### 6.1. Respiratory Challenges

The optimum respiratory pathway is a major determinant in the choice and outcome of anaesthesia. The lung in SCD patients is a major target for acute and chronic complications; this usually worsens the hypoxia prevalent in these patients. According to the National Heart, Lung and Blood Institute, acute chest syndrome (ACS) occurs when sickle cells become trapped in the pulmonary vasculature blocking the flow of blood and oxygen to the lungs. ACS had been reported to occur following respiratory tract infections, administration of opioids, or excessive use of intravenous fluid used in treatment of vasoocclusive crises [52–54]. ACS causes pneumonia, fever, pain, and severe cough and may lead to permanent damage to the lungs. Cellular respiration is markedly reduced in SCD, and this is further worsened by ACS; hence, the need for preoperative treatment of ACS in SCD patients before undergoing kidney transplant surgery [55]. ACS had also been reported as a complication of general anaesthesia and surgery. In a series of 604 patients studied by Vichinsky and colleague ACS was reported to develop on average of 3 days after surgery and lasted for 8 days [56]. 

Chest infections and obstructive airway diseases not related to sickle cell vasoocclusion were independently associated with worse outcome in SCD patients following surgery, probably resulting from local or systemic hypoxia, increased sickle hemoglobin (Hb S) polymerization, and complications of anaesthesia which are common in these patients. Bone marrow infarction and necrosis is a known complication of SCD and occasionally leads to embolisation of necrotic debris and fat to the pulmonary vasculature [57]. Bronchial asthma and antecedent airways hyperreactivity are not a classical feature of SCD. However, hyperreactivity was reported following cold air challenge in 83% of asymptomatic SCD children with history of reactive airways [58]. This tells that SCD patients have to be evaluated for reactive obstructive airway disease and steps taken to prevent occurrence before, during, or after surgery.

About 30% of patients with sickle cell disease were reported to have pulmonary hypertension, which is a serious and potentially fatal condition. Pulmonary hypertension could result from sickle cell-related vasculopathy, chronic oxygen desaturation from sleep hypoventilation, pulmonary damage from recurrent ACS, repeated episodes of thromboembolism, or high pulmonary blood flow due to anemia [59, 60]. The development of pulmonary hypertension raises the risk for cor pulmonale, recurrent pulmonary thrombosis, and worsened hypoxemia, all of which increases the frequency and severity of vasoocclusive episodes and hence complications of surgery in SCD [60]. Phosphodiesterase inhibitor had been noted to reduce intrapulmonary vascular pressure. We reported remarkable improvement in pulmonary hypertension of our pretransplant sickle cell disease patient with ESRD in response to sildenafil, the patient later had successful kidney transplant [35].

### 6.2. Immunological Challenges

Patients with SCD had been reported to have impairment in their immune status. This could result from both direct and indirect negative impacts on the immune system. Sickle cells crises especially vasoocclusive and sequestration crises had been associated with decreased blood flow to various tissues with antecedent reduction in tissue perfusion and delivery of immune cells to target site. For reasons, not clearly defined, the immune cells in SCD patients had also been noted to have variable impairment in their innate functions and this had led to increased infections, skin ulcer, and slowed wound healing [55]. Furthermore, in sickle cell disease patients, the spleen usually becomes atrophic and nonfunctional in adolescents and adults as a result of recurrent infarction. Also, patients with SCD may undergo therapeutic splenectomy following hypersplenism and/or recalcitrant sequestration crises. The spleen plays a major role in immunoregulation; thus, this auto- or iatrogenic splenectomy had been associated with significant immunodeficiency. 

Therapeutic immunosuppression is a cardinal component of management of kidney transplant, this however poses a potential challenge in curtailing infection in SCD patients following kidney transplant. However, adequate prophylactic measures including use of appropriate pre- and postexposure vaccination, antibiotics, and strict safety precautions had been associated with optimal control of infection in these patients. Further measures in control of infection in these patients include barrier nursing, intensive post op management, strict personal and environmental hygiene, drug monitoring, surveillance and screening for culprit infections and malignancies, early therapeutic interventions, and infection control. 

Patients with SCD are prone to multiple blood transfusions because of the prevalent perennial anaemia and recurrent crises. This exposes them to increasing incidence of alloimmunization to red blood cell, white blood cell, platelet, protein and human leucocyte antigens [61]. Thus SCD patients are potentially more likely to develop various forms of cellular and humoral immunoreactions including organ rejection, thus posing a challenge in donor/organ selection [62]. However, reports of hyperacute, acute, and chronic rejection in patients with SCD following kidney transplant were not significantly different from non-SCD patients [3, 5, 31]. This had been attributed to various precautionary measures including limiting transfusion to when it is necessary, scrupulous screening for antibodies, typing and strict matching of donor and recipient, plasmapheresis, leukodepletion, induction immunosuppression, and appropriate immunosuppressive therapy [63].

## 7. Other Challenges

Older children and adult patients with sickle cell disease are subject to other medical problems, including impaired physical development, hepatic, gall bladder and gum diseases, and, in severe cases, multiple-organ failure. These conditions have potentials to pose challenges in preoperative, operative, and postoperative management of these patients during kidney transplant.

Patients with sickle cell disease requiring kidney transplant are also prone to challenges experienced by their normal counterpart. This varies from universal challenges like scarcity of donor to challenges peculiar to resource poor nations like poverty, ignorance, lack of facilities, and personnel [2].

In conclusion, kidney transplant in patients with sickle cell nephropathy is plagued with multiplicity of challenges; however, outcome of few kidney transplant done so far in these patients is encouraging. There is a need for multidisciplinary and subspecialty coordination in the management of patients with sickle cell nephropathy requiring kidney transplant. 

## Figures and Tables

**Table 1:** Outcomes of kidney transplant in sickle cell disease patient.

Author	outcome
Chatterjee (1980) [24]	1-year patient survival 86% 1-year graft survival 67% mortality 1st year 13% Increased sickle cell crisis
Barber et al. (1987) [22]	1-year graft survival 25% Crises in 75%
Miner et al. (1987) [27]	Recurrent nephropathy
Chatterjee (1987) [25]	1-year patient survival 88% 1-year graft survival 82% in live, 62% in cadaveric, Increased sickle cell crisis
Montgomery et al. (1994) [23]	1-year patient and graft survival 100% Increased sickle cell crisis
Warady and Sullivan (1998) [29]	1-year graft survival 89% 2-year graft survival 71% Patient survival 89%
Ojo et al. (1999) [31]	Delayed function 24% Acute rejection 26% 1-year graft survival 78% 3-year graft survival 46% 1-year patient survival 78% 3-year patient survival 59% Median survival 33 months Recurrent nephropathy
Bleyer et al. (2001) [28]	1-year patient survival 90.5% 3-year patient survival 75.0% 1-year graft 82.5% 3-year graft survival 53.8%
Abbott et al. (2002) [8]	Risk of mortality same as in transplanted non sickle cell disease
O'Rourke et al. (2008) [32]	Recurrent allograft dysfunction from vascular congestion and tubular necrosis
Scheinman (2009) [33]	10-year patient survival 56%
Kim et al. (2011) [34]	Intragraft sickle cell vasoocclusive crisis as a cause of early allograft loss

## References

[1] Platt OS, Brambilla DJ, Rosse WF (1994). Mortality in sickle cell disease. Life expectancy and risk factors for early death. *The New England Journal of Medicine*.

[2] Okafor UH, Ekwem I, Wokoma FS (2012). Challenges of kidney care in a resource poor nation: a study of private kidney care centre in Nigeria. *Nigerian Medical Journal*.

[3] Status van Eps LW

[4] Derebail VK, Nachman PH, Key NS, Ansede H, Falk RJ, Kshirsagar AV (2010). High prevalence of sickle cell trait in African Americans with ESRD. *Journal of the American Society of Nephrology*.

[5] François L, Nadjib H, Katia SS (2012). Hemoglobin SC disease complications: a clinical study of 179 cases. *Haematologica*.

[6] Falk RJ, Scheinman JL, Phillips GI, Orringer E, Johnson A, Jennette JC (1992). Prevalence and pathologic features of sickle cell nephropathy and response to inhibition of angiotensin-converting enzyme. *The New England Journal of Medicine*.

[7] Scheinman JI (2006). Tools to detect and modify sickle cell nephropathy. *Kidney International*.

[8] Abbott KC, Hypolite IO, Agodoa LY (2002). Sickle cell nephropathy at end-stage renal disease in the United States: patient characteristics and survival. *Clinical Nephrology*.

[9] Bolarinwa RA, Akinlade KS, Kuti MAO, Olawale OO, Akinola NO (2012). Renal disease in adult Nigerians with sickle cell anaemia: a report of prevalence, clinical features and risk features. *Saudi Journal of Kidney Diseases and Transplantation*.

[10] Pham PTT, Pham PCT, Wilkinson AH, Lew SQ (2000). Renal abnormalities in sickle cell disease. *Kidney International*.

[11] Etteldorf JN, Tuttle AH, Clayton GW (1952). Renal function studies in pediatrics. *American Journal of Diseases of Children*.

[12] Morgan AG, Serjeant GR (1981). Renal function in patients over 40 with homozygous sickle-cell disease. *British Medical Journal*.

[13] De Jong PE, Statius van Eps LW (1985). Sickle cell nephropathy: new insights into its pathophysiology. *Kidney International*.

[14] Alleyne GAO, Statius van Eps LW, Addac SK, Nicholson GD, Schouten H (1975). The kidney in sickle cell anemia. *Kidney International*.

[15] Statius van Eps LW, De Jong PE, Schrier RW, Gottschalk C (1997). Sickle cell disease. *Diseases of the Kidney*.

[16] Allon M (1990). Renal abnormalities in sickle cell disease. *Archives of Internal Medicine*.

[17] Vaamonde CA (1984). Renal papillary necrosis in sickle cell hemoglobinopathies. *Seminars in Nephrology*.

[18] Serjeant GR (1992). *Sickle Cell Disease*.

[19] Foucan L (1998). A randomized trial of captopril for microalbuminuria in normotensive adults with sickle cell anemia. *American Journal of Medicine*.

[20] Powars DR, Elliott-Mills DD, Chan L (1991). Chronic renal failure in sickle cell disease: risk factors, clinical course, and mortality. *Annals of Internal Medicine*.

[21] Port FK, Nissenson AR (1989). Outcome of end-stage renal disease in patients with rare causes of renal failure. II. Renal or systemic neoplasms. *Quarterly Journal of Medicine*.

[22] Barber WH, Deierhol MH, Julian BA (1987). Renal transplantation in sickle cell anaemia and sickle cell trait. *Clinical Transplantation*.

[23] Montgomery R, Zibari G, Hill GS, Ratner LE (1994). Renal transplantation in patients with sickle cell nephropathy. *Transplantation*.

[24] Chatterjee SN (1980). National study on natural history of renal allografts in sickle cell disease or trait. *Nephron*.

[25] Chatterjee SN (1987). National study in natural history of renal allografts in sickle cell disease or trait: a second report. *Transplantation Proceedings*.

[26] NIH publications (2002). *The Management of Sickle Cell Disease: Renal Abnormalities in Sickle Cell Disease*.

[27] Miner DJ, Jorkasky DK, Perloff LJ, Grossman RA, Tomaszewski JE (1987). Recurrent sickle cell nephropathy in a transplanted kidney. *American Journal of Kidney Diseases*.

[28] Bleyer AJ, Donaldson LA, McIntosh M, Adams PL (2001). Relationship between underlying renal disease and renal transplantation outcome. *American Journal of Kidney Diseases*.

[29] Warady BA, Sullivan EK (1998). Renal transplantation in children with sickle cell disease: a report of the North American Pediatric Renal Transplant Cooperative Study (NAPRTCS). *Pediatric Transplantation*.

[30] Spector D, Zachary JB, Sterioff S, Millan J (1978). Painful crises following renal transplantation in sickle cell anemia. *American Journal of Medicine*.

[31] Ojo AO, Govaerts TC, Schmouder RL (1999). Renal transplantation in end-stage sickle cell nephropathy. *Transplantation*.

[32] O'Rourke EJ, Laing CM, Khan AU (2008). The case | Allograft dysfunction in a patient with sickle cell disease. *Kidney International*.

[33] Scheinman JI (2009). Sickle cell disease and the kidney. *Nature Clinical Practice Nephrology*.

[34] Kim L, Garfinkel MR, Chang A, Kadambi PV, Meehan SM (2011). Intragraft vascular occlusive sickle crisis with early renal allograft loss in occult sickle cell trait. *Human Pathology*.

[35] Okafor UH, Wachukwu C, Emem-Chioma P, Wokoma FS (2012). Kidney transplant in a 26 year old Nigerian patient with sickle cell nephropathy. *Case Reports in Nephrology*.

[36] Brennan DC, Lippmann BJ, Shenoy S, Lowell JA, Howard TK, Flye MW (1995). Living unrelated renal transplantation for sickle cell nephropathy. *Transplantation*.

[37] Scheinman JI, Avner ED, Harmon WE, Niaudet P, Yoshikawa N (2004). Sickle cell nephropathy. *Paediatric Neprhology*.

[38] Janik J, Seeler RA (1980). Perioperative management of children with sickle hemoglobinopathy. *Journal of Pediatric Surgery*.

[39] Rutledge R, Croom RD, Davis JW (1986). Cholelithiasis in sickle cell anemia: surgical considerations. *Southern Medical Journal*.

[40] Gibson JR (1987). Anesthesia for sickle cell diseases and other hemoglobinopathies. *Sem Anesthesia*.

[41] Ware R, Filston HC, Schultz WH, Kinney TR (1988). Elective cholecystectomy in children with sickle hemoglobinopathies. Successful outcome using a preoperative transfusion regimen. *Annals of Surgery*.

[42] Esseltine DW, Baxter MRN, Bevan JC (1988). Sickle cell states and the anaesthetist. *Canadian Journal of Anaesthesia*.

[43] Bihl G (2002). Kidney transplantation in a patient with sickle cell kidney disease?. *Medscape Transplantation*.

[44] Donnelly PK, Edmunds ME, O'Reilly K (1988). Renal transplantation in sickle cell disease. *The Lancet*.

[45] NIH Publications (2002). *The Management of Sickle Cell Disease: Anaesthesia and Surgery*.

[46] Martin H, Steinberg MD (1999). Management of sickle cell disease. *The New England Journal of Medicine*.

[47] Haywood LJ (2009). Cardiovascular function and dysfunction in sickle cell anemia. *Journal of the National Medical Association*.

[48] Covitz W, Espeland M, Gallagher D, Hellenbrand W, Leff S, Talner N (1995). The heart in sickle cell anemia: the cooperative study of sickle cell disease (CSSCD). *Chest*.

[49] Tsironi M, Aessopos A (2005). The heart in sickle cell disease. *Acta Cardiologica*.

[50] Leight L, Snider TH, Clifford GO, Hellems HK (1954). Hemodynamic studies in sickle cell anemia. *Circulation*.

[51] Gray A, Anionwu EN, Davies SC, Brozovic M (1991). Patterns of mortality in sickle cell disease in the United Kingdom. *Journal of Clinical Pathology*.

[52] Miller ST, Sleeper LA, Pegelow CH (2000). Prediction of adverse outcomes in children with sickle cell disease. *The New England Journal of Medicine*.

[53] Vichinsky EP, Neumayr LD, Earles AN (2000). Causes and outcomes of the acute chest syndrome in sickle cell disease. *The New England Journal of Medicine*.

[54] http://www.livestrong.com/.

[55] Milner PF, Brown M (1982). Bone marrow infarction in sickle cell anemia: correlation with hematologic profiles. *Blood*.

[56] Vichinsky EP, Haberkern CM, Neumayr L (1995). A comparison of conservative and aggressive transfusion regimens in the perioperative management of sickle cell disease. *The New England Journal of Medicine*.

[57] Leong MA, Dampier C, Varlotta L, Allen JL (1997). Airway hyperreactivity in children with sickle cell disease. *Journal of Pediatrics*.

[58] Samuels MP, Stebbens VA, Davies SC, Picton-Jones E, Southall DP (1992). Sleep related upper airway obstruction and hypoxaemia in sickle cell disease. *Archives of Disease in Childhood*.

[59] Powars D, Weidman JA, Odom-Maryon T, Niland JC, Johnson C (1988). Sickle cell chronic lung disease: prior morbidity and the risk of pulmonary failure. *Medicine*.

[60] Rosse WF, Gallagher D, Kinney TR (1990). Transfusion and alloimmunization in sickle cell disease. *Blood*.

[61] King KE, Shirey RS, Lankiewicz MW, Young-Ramsaran J, Ness PM (1997). Delayed hemolytic transfusion reactions in sickle cell disease: simultaneous destruction of recipients' red cells. *Transfusion*.

[62] Sullivan KM, Agura E, Anasetti C (1991). Chronic graft-versus-host disease and other late complications of bone marrow transplantation. *Seminars in Hematology*.

[63] Jay N, James M, Andrew B, Simon B, Andrew RR, Nicholas GI (2013). Sickle cell and renal transplant: a national survey and literature review. *Experimental and Clinical Transplantation*.

